# State of the Art on Biomaterials for Soft Tissue Augmentation in the Oral Cavity. Part II: Synthetic Polymers-Based Biomaterials

**DOI:** 10.3390/polym12081845

**Published:** 2020-08-17

**Authors:** Manuel Toledano, Manuel Toledano-Osorio, Álvaro Carrasco-Carmona, Cristina Vallecillo, Raquel Toledano, Antonio Luis Medina-Castillo, Raquel Osorio

**Affiliations:** 1 Dental Materials Section, Faculty of Dentistry, University of Granada, Colegio Máximo de Cartuja s/n, 18071 Granada, Spain; toledano@ugr.es (M.T.); alcarcar94@correo.ugr.es (Á.C.-C.); cvallecillorivas@hotmail.com (C.V.); rtoleosorio@gmail.com (R.T.); rosorio@ugr.es (R.O.); 2 NanoMyP Spin-Off University of Granada Enterprise, BIC Building, Office 235 and Lab 121. Av. Innovación 1 E-18016, 18100 Granada, Spain

**Keywords:** polymer, soft tissue, oral, hydrogel, synthetic, review, augmentation

## Abstract

Most of the polymers used as biomaterials for scaffolds are naturally occurring, synthetic biodegradable, and synthetic non-biodegradable polymers. Since synthetic polymers can be adapted for obtaining singular desired characteristics by applying various fabrication techniques, their use has increased in the biomedical field, in dentistry in particular. The manufacturing methods of these new structures include many processes, such as electrospinning, 3D printing, or the use of computer-aided design/computer-aided manufacturing (CAD/CAM). Synthetic polymers show several drawbacks that can limit their use in clinical applications, such as the lack of cellular recognition, biodegradability, and biocompatibility. Moreover, concerning biodegradable polymers, the time for matrix resorption is not predictable, and non-resorbable matrices are preferred for soft tissue augmentation in the oral cavity. This review aimed to determine a new biomaterial to offset the present shortcomings in the oral environment. Researchers have recently proposed a novel non-resorbable composite membrane manufactured via electrospinning that has allowed obtaining remarkable in vivo outcomes concerning angiogenesis and immunomodulation throughout the polarization of macrophages. A prototype of the protocol for in vitro and in vivo experimentation with hydrogels is explained in order to encourage innovation into the development of promising biomaterials for soft tissue augmentation in the near future.

## 1. Natural versus Synthetic Polymers

In tissue engineering, one of the most important biomaterials for scaffolds is the polymeric matrices [1]. They are attracting a great deal of attention because of their unique properties, which include a high porosity with a very small pore size, great surface-to-volume ratio, and biodegradation. It has also been demonstrated that they provide varying surface chemistry, interconnected porosity and surface area, and unique geometries for a direct regeneration of the tissue [2]. The main types of polymers used as biomaterials are naturally occurring polymers, synthetic biodegradable polymers, and synthetic non-biodegradable polymers [2]. Considering their nature, they can be natural polymers (collagen, silk, gelatin, and fibrin glue), polynucleotides, and polysaccharides (hyaluronic acid, chitosan) [3]. The scaffold will degrade over time and be replaced by a new extracellular matrix (ECM) secreted by fibroblasts [4]. When compared with synthetic matrices, natural polymers exhibit some advantages, including an enhanced interaction with host cells and a greater biocompatibility. In contrast, collagen-based scaffolds possess a recurrent wound contracture and a lack of biostability [2]. The combination of these polymers with other ECM molecules or the cross-linking of collagen matrices have offset these shortcomings. On the other hand, the use of synthetic materials has been raised because they can be adapted for obtaining singular desired characteristics by applying various fabrication techniques, and their use has increased in the biomedical field. In addition, using synthetic membranes diminishes the potential risk of disease transmission and immunogenic response. In tissue engineering, some of the most used synthetic polymers are polycaprolactone (PCL), poly(lactic acid) or polylactic acid or polylactide (PLA), and poly(lactic-co-glycolic) acid (PLGA) [5,6].

## 2. Manufacturing Processes of Synthetic Matrices for Oral Soft Tissue Augmentation

Numerous efforts have been made in the last four decades for the treatment of periodontal apparatus and the regeneration of its surrounding soft tissue areas instead of repair. These attempts include barrier membranes, root surface conditioning, graft materials, growth factors, and gene therapy [7]. Nevertheless, these approaches still exhibit significant clinical disadvantages; autologous grafts availability is scarce, gene therapy is still involved in the triggering of tumor appearances or host immune reactions, biomaterials are associated with a high failure rate, and growth factors are unstable. Therefore, there is a significant need for high efficacy and highly efficient treatments, paving the way for periodontal tissues restoration [8]. These past decades have witnessed a rise in interest in the creation of biomimetic or bioinspired regenerative materials due to the advancements in the field of nanomaterials. The appearance of new techniques have allowed modulating the composition, size, dimension, microstructure, morphology, and form of these structures and have facilitated the creation of multiple biomaterials tissue engineering centers in the regeneration of tissues via a combination of cells with bioactive factors and scaffolds. It has been applied to soft tissue generation and augmentation. Over the years, tissue engineering has progressed significantly. The appearance of advanced processing technologies and a third generation of biomaterials have allowed the transformation of the manufacturing concept. This has resulted in the production of scaffolds with tailored properties to be applied in difficult situations, such as functional, esthetic, or load-bearing ones [8]. The manufacturing methods of these new structures include distinct processes, such as electrospinning, 3D printing, or the use of computer-aided design/computer-aided manufacturing (CAD/CAM), which, at the moment, are the three methods that are the most referred to in the literature. Specifically, electrospinning has been used for oral applications of biomaterials with success in both in vitro and in vivo research [9,10,11].

### 2.1. Electrospinning

**Figure 1:**
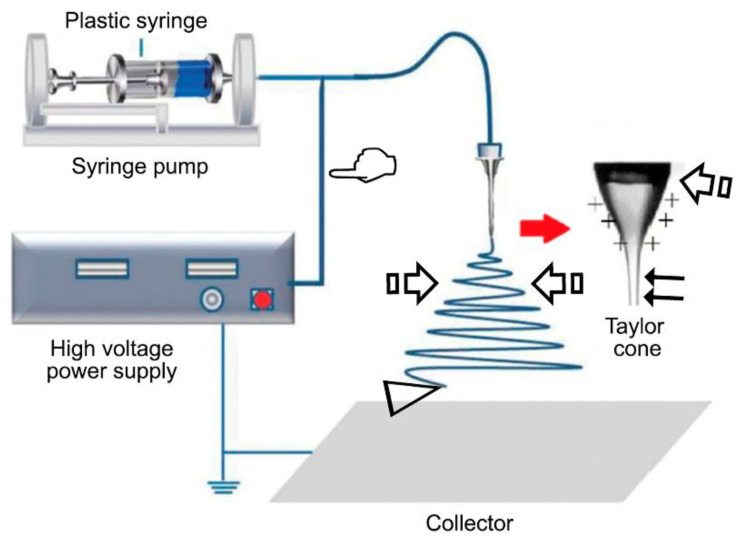
This technique begins with the application of a high potential (14–16 kV) between the metal collector, located at a predetermined distance from the tip of the needle tip, and the polymer solution droplet in the syringe (pointer). The electrostatic force turns the drop into a hemispherical form, which is known as the Taylor cone (arrow). When the electrostatic potential difference is high enough to overcome the surface tension of the polymer drop located at the tip of the metal needle, an elongated cone is generated. This results in the production of a charged liquid jet from the Taylor cone, which is able to travel through a linear distance [generally 1–2 cm, known as the jet length (double arrows)]. The charged liquid jet is submitted to whipping instabilities. These alterations cause the jet to acquire a longer and thinner form due to the plastic deformation, stretching its length thousands of times its original size. The plastic deformation tends to dry the jet, and it results in ultrafine fibers before their arrival to the metallic collector (faced arrows). The solvents are evaporated on the way from the needle to the collector (arrow head) [12,15,18,19]. Figure reproduced and adapted from Chen et al. (2019) [20]. Copyright MDPI, 2019.

Several techniques have been employed for the synthesis of biomaterials by combining natural and/or synthetic polymers, including electrospinning [12]. First introduced in 1938 by Formhals [12] and popularized in the 1980s due to the increased interest in nanotechnology [13], electrospinning has since been defined as a versatile approach to create fibrous scaffolds for tissue regeneration [14]. This technique has received great attention due to its potential to create ultrafine fibers with various properties by applying an electrostatic field [15]. These fibers can be produced with a diameter range from 3 nm to 10 µm [16], while also exhibiting a high porosity, surface area to volume ratio, and flexibility [15]. An electrospinning apparatus is mainly comprised of four components: a source of high voltage (1–30 kV), a metallic needle or capillary, a grounded conductive collector (which can be a rotating drum or a flat plate), and a syringe pump [17] (Figure 1).

As many factors can influence the result, this process is defined as a complex process. This is positive for the platform technology, as its properties can be modified, such as the fiber morphology and diameter, but it also requires a high understanding of each method in order to allow their reproducibility [18]. Three main factors can affect the electrospinning procedure: the actual electrospinning (the electric field applied, the distance between the collector and the needle, the needle diameter, and the flow rate), solution (the solvent, viscosity, and conduction of the solution, and the polymer concentration) and environmental parameters (humidity and temperature) [21]. Each parameter can affect the electrospinning process and the resultant structure. However, it has been observed that after dissolving in an appropriate solution, almost all polymeric blends can be electrospun in a range of 7–30 kV [15]. The production of nanoscale fibers requires the application of a higher electrostatic field [15] and a favorable solution conductivity [21]. Increasing the conductivity will both increase the surface charge and decrease the fiber diameter [22]. When this parameter surpasses the critical value, it will hinder the Taylor cone formation, as its formation is determined by the electrostatic field of the surface charges created by the external electric field. An ideal dielectric polymer solution will not have enough charges to move onto the surface of the fluid, while a conductive solution will possess enough free charges to shift to the surface. This would allow the formation of the Taylor cone and the beginning of the electrospinning process [21]. 

Electrospinning allows the formation of nanostructured materials that mimic extracellular matrix (ECM) morphology. Electrospun nanofibers have been applied in a wide array of medical applications, such as wound dressing, tissue engineering scaffolds, drug delivery, and dentistry [23]. This technique has also been applied for the regeneration of soft tissue [24]. Every soft tissue presents a unique microstructure for a specific function, but they all include the same basic components; cells and ECM, which consists mainly of collagen, elastin, and other macromolecules such as glycoproteins. ECM is involved in cytodifferentiation and organogenesis, and it can be considered a scaffold that allows an organized repair after injuries [25]. Electrospun nanofiber matrices exhibit similar morphological characteristics to the ECM, so these scaffolds have been applied in the regeneration of various soft tissues, including non-connective (vascular, muscle, and neural tissue) and connective (ligament, skin, and tendon) tissues [26]. 

Electrospinning has demonstrated its potential to produce nanofibrous biomaterials, which in turn has proven its ability to promote tissue regeneration. This technique has improved more in the last decade than ever before [27]. All in all, electrospinning has achieved considerable success. Some recent developments include the appearance of new processes and methods to obtain more complex architectures such as mesh composition, modification of the setup for better fiber orientation, control of blending, and targeted fiber collection [28]. These new methodologies have allowed a better and more uniform cell distribution in the scaffolds, but the manufacturing procedure still requires a higher control of the fiber synthesis process and scaffold morphology and composition in order to be more reproducible [28]. Furthermore, Tebyetekerwa and Ramakrishna (2020) [29] highlighted that electrospinning still needs to improve in the near future. The next wave of electrospun biomaterials requires a better understanding of the nanofiber formation and created nanofibers in order to obtain a new type of nanofiber and the synthesis of new materials for a wide range of applications. Despite these challenges, electrospinning is still defined as a versatile technique, and when combined with innovative approaches, it is sure to have an impact in the field of tissue engineering.

### 2.2. 3D Printing

**Figure 2:**
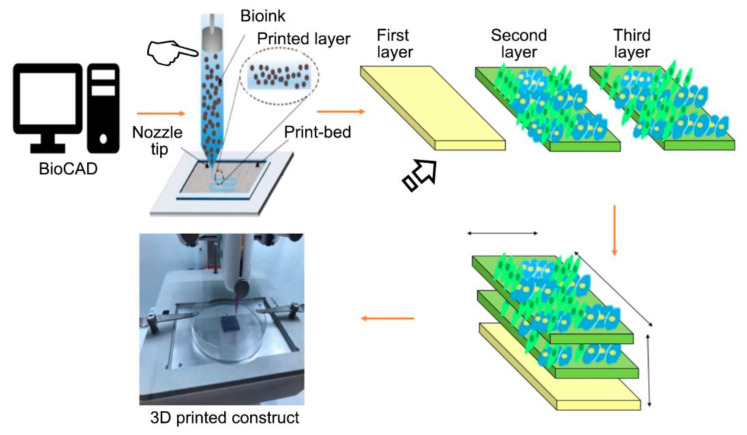
The most important part of this printing technique is the bioink (pointer), which is the matrix material that provides a proper environment for the transmission of cues and signals for the tissue formation and cellular function. To print 3D soft tissue-like structures, cells are either mixed with bioink and printed or printed separately as cell suspensions onto bioink. The 3D construct is assembled in an additive manner by printing layer by layer [34] (arrow). Figure reproduced and adapted from Hafezi et al. (2020) [36]. Copyright MDPI, 2020.

Tissue engineering is based on a combination of technologies and principles of different disciplines to obtain biomimetic three-dimensional (3D) porous scaffolds for guided tissue regeneration [30]. The tissue printing technology has been defined as an alternative that offers the possibility of generating biologic structures that enable tissue regeneration and function restoration [31]. This technology has led to the regeneration of tissues and organs, discarding the need for any tissue graft or mechanical device [32]. The regeneration is caused by the role of the scaffolds in cellular attachment and proliferation, vascularization, and nutrients transportation [33]. Organ-like tissue models need to exhibit a certain degree of complexity to reflect the in vivo situation as closely as possible. With respect to this highly demanded complexity, bioprinting shows a great potential to produce artificial 3D tissues and organs [34]. The technique is based on the synthesis of structures by printing cells along with matrix components in an organized and defined way, in a specific location and environment and in an appropriate number [35]. The advantage of this technology compared to standard tissue-engineering approaches is the exact positioning of cells and bioactives, such as signaling factors and matrix components (so-called bioinks), to obtain spatial control [34] (Figure 2).

Among these matrix materials, various components of the extracellular matrix (ECM), including collagen and fibrin, have been employed as bioink, but the matrix native structure is difficult to replicate [35]. The direct printing of cells in the bioink (described as 3D bioprinting) generates a specialized and compartmentalized construct with enhanced regenerative potential. This structure can deliver multiple cell types in a spatially accurate way. While bioink captures the cells, the bioprinting system deposits strands in a specific location, allowing the printing of spatial and reproducible cell scaffolds [30]. In order to allow this matter, the bioink must exhibit a certain viscosity to flow under shear stress and to turn solid after its extrusion. The difficulty of balancing cytocompatibility and printability is one of cell printing’s main challenges [37]. Therefore, bioink must exhibit good flow behavior, a high cell affinity, and should be able to retain its shape [30]. However, no single bioprinting technique is able to fabricate large-scale tissues [38].

3D printing was first used in 1986, and various materials have since been employed for bioprinting, including polymers [33,39]. This 3D printing has been used in a wide number of tissue engineering and regenerative medicine applications including cardiovascular, neural, cartilage, liver, tracheal engineering, skin, and soft tissue augmentation in the oral cavity. The resulting scaffold must exhibit proper porosity, cellular behavior, and mechanical properties, and they also must be biocompatible and possess a certain biodegradation degree [33]. The organization and composition of the environment are crucial to mediate cellular responses that affect the commitment and differentiation of stem cells, both in vivo and in vitro [35]. The printing process is formed by three stages: pre-processing, processing, and post-processing. In the first stage, details from the structure are acquired and transferred to a computer-aided design (CAD) system, in which the assemblage will be evaluated and customized before the scaffold construction. In the processing stage, the components are selected, which led to the production of new tissues. In the post-processing stage, the tissue is optimized and prepared for its introduction in the patient to alleviate undesired outcomes [33,40]. As biomaterial-cell interactions are crucial to cell proliferation and differentiation, the control of the biomaterials characteristics has to be considered. The inclusion of CAD software has allowed the production of controlled macro-, micro-, and nanoarchitecture of biomedical devices [41], which have improved the outcome of the printed scaffolds.

However, 3D application in regenerative dentistry (particularly periodontal regeneration) is still in its first steps [30]. The main 3D printing challenge, particularly in soft tissue applications, is that softer materials tend to gelatinize, even under small temperature changes. This can lead to the loss of scaffold structural integrity [40]. Fischer et al. (2016) [42] investigated 3D printing in cold environments, obtaining a minimal damage for the scaffold cohesion and allowing soft-tissue scaffolds biocompatibility. 3D scaffold printing has achieved considerable progress, but it still presents several drawbacks [41]. Selecting a suitable scaffold or biomaterial for tissue engineering and cell- and growth factor-doped scaffolds that still possess a low viability and stability poses a challenge. The release of substances immobilized on the scaffold still does not achieve the desired effects. There are also some challenges in the 3D printing synthesis parameters that need to be solved; however, if they are, this technique could improve tissue regeneration and lead to better clinical results. The combination of different printing techniques is also being studied to mimic biological architecture and functionality and to reduce production time. However, there is still room for improvement regarding inks and bioinks and their properties for printing and clinical applications. The improvement of supramolecular hydrogels is helping the development of advanced biomaterial inks and bioinks [43].

### 2.3. CAD/CAM

**Figure 3:**
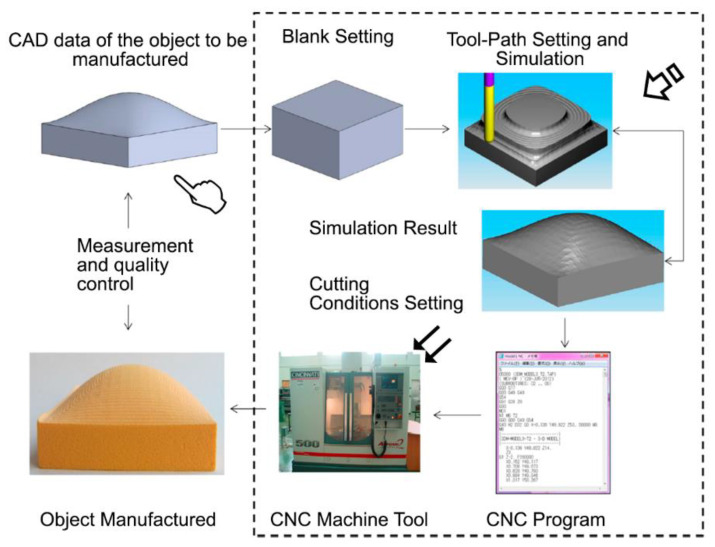
The computer-aided design/computer-aided manufacturing (CAD/CAM) systems are comprised of three main parts: (1) a data acquisition unit, which gathers information from the structure by using scanners, including the defect section and its surrounding and opposing structures, and transforms them to virtual data (pointer); (2) the software required for the data curation and model design (arrow); and (3) a device to manufacture the restoration using specific materials (double arrows) [45,46]. Figure reproduced and adapted from Ullah and Harib (2018) [47]. Copyright MDPI, 2018.

Technology based on computer-aided design/computer-aided manufacturing (CAD/CAM) has been employed for the design and manufacturing of products using digital technologies [44]. The main goal of this technology is to transform existing CAD data applying CAM manufacturing procedures following a basic pattern [44]. (1) The first step is the creation of a CAD model, in which specific software creates a 3D model of the object. (2) The second step involves conversion of the 3D model into a file in the *stl* format. STL files have become the standard format for this process. This file represents the 3D structure with a combination of triangles, storing vertices coordinates and the directions of each one. (3) The third step is slicing the STL model into thin layers through a *xy* plane. All layers are built on the previous one across the *z*-axis. (4) The fourth step is building the structure using the STL file slices. (5) The fifth step includes the selection of the required technology and materials to produce the structure. The composition of this infrastructure is shown in Figure 3.

CAD/CAM was developed originally for its use in the automobile and aircraft industries [48], but the fast evolution of these systems led to their introduction in medicine due to their potential to produce medical devices, orthopedic implants, and artificial tissues and organs [49]. Among their medical applications, CAD/CAM has been greatly applied in dentistry, with many researchers using CAD/CAM for diagnosis and treatment planning and maxillofacial implants, dentures, crowns, and tooth tissue engineering [49,50,51]. A reliable and successful CAD/CAM system should be able to combine different materials to produce high-quality restorations [52]. This technology is attractive because of its use of digital impressions, which makes the procedure more comfortable for patients, as well as a more economic process that uses less working time and more aesthetic and biocompatible materials, making it overall a more appealing approach that promotes business and improves ecofriendly dentistry [53]. CAD/CAM systems are closely related to three-dimensional printing (3D printing), and while the firsts are based on a subtractive process (the desired products are obtained from an initial block object), 3D printing is based on an additive process (the object is built upon in a layer-by-layer approach). In 3D printing, only alloplastic materials can be used, but CAD/CAM can be applied on many different block grafts, including alloplastic, allogeneic, and xenogeneic grafting materials. In contrast, despite CAD/CAM offering customization of the structure shape and surface topography, 3D printing can also modify the internal architecture [54].

As previously mentioned, CAD/CAM has been more extensively applied in the dentistry field. In implantology, concretely in the aesthetic sector, one of the key factors for long-term implant success is the soft tissue stability [55]. The relationship between CAD/CAM custom abutments and soft tissue has been studied, with supports offering favorable results in terms of the tissue stability [56,57]. Proussaefs (2016) [58] applied this technology in edentulous patients. A CAD/CAM polymethylmethacrylate impression was utilized in order to imitate the soft tissue anatomy before the fabrication of the definitive prosthesis, obtaining identical contours to the original structure. They theorized that the transference of the soft tissue architecture could be beneficial because it was synthesized exactly as the interim prosthesis, and so, the pressure applied was minimal or non-existent from the surrounding area. This technique also offered the option of contouring the soft tissue to accommodate the definitive prosthesis. CAD/CAM procedures have also been applied for soft tissue regeneration. Zopf et al. (2014) [59] created a biomimetic scaffold that consisted of polycaprolactone impregnated with chrondrogenic growth factors in a hyaluronic acid/collagen hydrogel for use in nasal and auricular reconstruction. These results suggest that CAD/CAM techniques can be applied to obtain better soft dental tissue stabilization, but their extrapolation to other kind of tissues has yet to be thoroughly examined. In recent years, the interest in CAD/CAM and 3D printing techniques has substantially increased, which has in turn transformed the regenerative medicine and tissue engineering fields. These advances are currently leading to new horizons, such as the possibility of manufacturing specific scaffolds or constructs with defined properties for each patient [43].

## 3. New Trends in Synthetic Polymeric Blends for Oral Tissue Engineering

### 3.1. Resorbable versus Non-Resorbable Matrices

Synthetic scaffolds can be manufactured in greater amounts and exhibit a longer shelf life than their natural equivalents [3]. Furthermore, they possess consistent properties, which include the degradation rate, elastic modulus, and tensile strength. However, there are several drawbacks that can prevent their use in clinical applications, such as the lack of cellular recognition, biodegradability, and biocompatibility [6]. In the current market, the majority of resorbable synthetic polymer membranes consist of aliphatic polyesters, including poly(ε-caprolactone) (PCL), poly(glycolic acid) (PGA), poly(lactic acid) (PLA), poly(hydroxyl butyric acid), and poly(hydroxyl valeric acid) and their copolymers. However, most of these matrices are subjected to major disadvantages, including the presence of inflammatory reactions derived from the production of their degradation products. It has also been observed that PLA and PGA membranes produce a decreased defect fill when compared to non-resorbable membranes. They are also less biologically active than their natural counterparts. Still, their controllable biodegradability, manageability, processability, low rigidity, and excellent biocompatibility have promoted their use in the clinic environment and theoretical experiments for biomedical applications, particularly in guided tissue regeneration [60].

As it was stated in Part I of the present study [61], there is a lack of appropriate mechanical strength and degradation profiles in natural polymers, while synthetic ones are considered biologically inert. Previous investigations have stated that some polyester-based membranes increase their stiffness and fragility when they are placed in phosphate buffered saline (PBS) or artificial saliva solution. Nowadays, the development of membranes that exhibit a predictable degradation rate, adequate mechanical properties, and structures that mimic the native extracellular environment is still a challenge. The combination of two kinds or more polymers in order to minimize their limitations and obtain better synergistic effects has been defined as an efficient solution [60]. On the other hand, with the constant assembly of new connective tissue and degradation of the former matrix, the material may become gradually substituted or incorporated by host tissues [62] without predictability. This poses a drawback. In order to avoid it and to improve the mechanical properties, osteogenic, cell-membrane interactions, and hydrophilicity, and to confer antibacterial properties, researchers have recently been proposed a novel non-resorbable composite membrane, manufactured via electrospinning with a mixture of (MMA)_1_-co-(HEMA)_1_ and (MA)_3_-co-(HEA)_2_ that has allowed obtaining remarkable outcomes [10,63].

### 3.2. Loading of Matrix Scaffolds Strategies to Stimulate the Formation of Capillary-Like Networks In Vivo Recruitment of Osteoblasts

Assessing angiogenesis through the test of the microvessel densities and proliferation densities of the vasculature when matrices are going to perform as soft tissue augmentation materials is of primary importance. In the absence of any biomaterial, the existing blood vessels grow with a spontaneous speed of several tenths of a micrometer per day. This is considered as too slow to allow an appropriate nutrient flow to the cells in the surgical area. The new capillaries can be defined as transient in nature and can require exogenous supplementation [64]. Hence, the development of new complementary strategies for angiogenesis stimulation are understood as vital to guarantee the survival of large tissue-engineered constructs [65]. In this line, Si^4+^ has been probed to induce angiogenesis and cell proliferation [66]. In addition, Si can stimulate collagen type I synthesis and human osteoblastic differentiation [67], which accommodate matrix-dependent gradients in O_2_ tensions [68]. Silica and zinc oxide have been demonstrated to display neovascularization [10,64]. Zn stimulates the angiogenic differentiation of stem cells [67]. Materials with proangiogenic potential are an alternative to the application of recombinant inductive growth factors [65]. Vascular morphogenetic proteins regulate the neovasculature/neoangiogenesis in newly formed tissues, particularly angiopoietins and vascular endothelial growth factors (VEGFs) [66]. In wound healing, VEGF stimulates angiogenesis, which implies that its expression is higher in early healing phases after augmentation. However, VEGF expression can be upregulated in hypervascularization processes of human connective tissues too [69]. 

Thereby, techniques involving growth factors such as the triggering of VEGFs are examples of successful approaches [64]. Nevertheless, the delivery of growth factors has revealed promising results in the promotion of angiogenesis in tissue regeneration. Despite this, it presents some limitations, including uncontrolled release, high costs, short half-life, and a requirement for high doses [66]. So, VEGF expression is induced by Si^4+^ by upregulating in endothelial cells nitric oxide synthase and nitric oxide production [66]. Recently, novel nanostructured membranes silica-loaded (Nm) and doped with zinc (Zn-Nm) or doxycycline (Dox-Nm) have been used. The regenerative potential has been tested in a critical-sized calvarial bone defect rabbit model [10]. It has been observed that the greatest vasculature value was attained after using Zn-Nm; when compared with Dox-Nm, it showed clear remodeling signs, based on nutrients supply and cells recruitment, as previously stated [70]. These remodeling strategies might be incorporated in future therapies for soft tissues augmentation. 

Osteoblasts, which were present in a higher amount in Zn-Nm and Dox-Nm-treated animals, secret VEGFs, while endothelial cells do the same with bone morphogenetic protein (BMP-2) for a combination of angiogenesis and osteogenesis [71]. In order to receive oxygen through diffusion, cells must be at a distance of 100–200 μm from blood vessels. If this distance is surpassed, blood vessels can arise by sprouting in response to hypoxic tissue-derived local cues. Growth and stabilization are two events underlying sprouting angiogenesis. Metalloproteinases (MMPs) release mediates the permeability and degradation enhancement of the basement membrane in the growth phase via vasodilation of the existing vessels [64]. Among MMP inhibitors, doxycycline and Zn have been described as potent ones [72], but the latter is also involved in the stimulation of angiogenic and osteogenic stem cells differentiation [67]. It is believed that MMPs inhibitory capacity could be offset by this potential. By inhibiting MMP activities, doxycycline could indirectly prevent the activation of cytokines such as transforming growth factor β (TGF-β), which could be associated with the BMP-2 signaling of collagen formation [73]. BMP-2 reduction has been proven when doxycycline was present [74,75]. In the various human body connective tissues, the most abundant protein is collagen. Specifically, collagen I is predominant in human connective tissues repair and scar tissue formation [69]. 

### 3.3. The Intertwined Concepts of Tissue Engineering and the Innate Immune Response Related with Angiogenesis Macrophages Functional Polarization (M1/M2)

Even though cell dimensions are comprised in a micrometer scale, their evolution in vivo is undertaken in close contact with the extracellular matrix, which is defined as a substratum whose structural and topographical features exhibit a nanometer scale. Hence, the development of nanostructures with properties similar to those of a cell’s natural environment could interact with them at a molecular level. This could allow an effective control of several tissue regeneration processes, including cell differentiation, proliferation, and adhesion [76]. The study of the cell population, as fibroblasts, osteoblasts, keratinocytes, and endothelial cells [61], is crucial to understand the role of implanted biomaterials at the host tissue. Osteal macrophages (osteomacs) participate in a key function: remodeling. One of the first cell types to make contact with implanted biomaterials is monocyte/macrophage lineage-derived cells. They can differentiate toward classical M1 (pro-inflammatory) or M2 macrophages (anti-inflammatory) or subsequently fuse into osteoclasts or multinucleated giant cells (MNGCs), which are related to biomaterial rejection [77]. It is necessary to reestablish a foreign body equilibrium to prevent further tissue loss [78]. In order to increase biocompatibility, it would be possible to modulate macrophages polarity from the pro-inflammatory M1 to anti-inflammatory M2 phenotype by, for instance, using DNA expressing interleukin (IL)-4 or 10 encapsulated in hyaluronic acid-poly(ethyleneimine) [79]. 

Soft tissue regeneration and further augmentation is a process that requires the collaboration of cells from different systems, and they are classified as complex procedures. Interdisciplinary immunology has allowed classifying the roles of indispensable immune cells in the progress of new tissue and outlining how an early immune environment is crucial for the outcome of the regeneration. This indicates the importance of immune response modulation. Monocyte/macrophage lineage cells may trigger a host foreign body response led by the implantation of matrices. Thus, immunomodulation manipulation has become a valuable instrument to guide the formation of soft tissue. Interferon gamma (IFN-γ) and bacterial endotoxins, such as lipopolysaccharide (LPS) and some chemokines, can classically activate macrophages, which can display the M1 phenotype characterized by a high pro-inflammatory mediator expression and both reactive oxygen and nitrogen intermediates production. In type I inflammatory responses, M1-polarized macrophages can act as effector or inducer cells. When macrophages are exposed to Th2-type cytokines, such as IL-4, IL-10, IL-13, or glucocorticoids hormones, they can activate alternatively a M2 profile that participates in and promotes angiogenesis and matrix remodeling. Soft tissue regeneration will be produced around materials if M2 macrophage polarization is favored.

In order to obtain a pattern of immunomodulation, several approaches have tried to obtain modifications of the biomaterials properties for a better moderation of the associated immunological reactions. Engineered-modified biomaterials generate specific immune microenvironments [71]. Fibrous tissue complies with inflammatory cells recruitment and inflammatory microenvironment presence [71]. Zn-Nm and Dox-Nm have inducted the lowest M1 counts in in vivo animal experimentation [10]. When the M1 population decreases, the matrix MMPs produced by M1 diminish. Zinc and doxycycline are inhibitors of MMPs [72], and thus, their pro-healing role is jeopardized. M2 counts were found to be higher in those samples that were treated with both Zn-Nm and Dox-Nm, and they have been demonstrated to promote vascular/matrix and angiogenesis remodeling [80], which corresponded with the highest vasculature attained among groups in the present research. Furthermore, M2 cells were predominant in the remodeling of tissue and immunoregulation processes [81]: they decreased phagocytic capability [82], and after using zinc and doxycycline, they strongly reduced the damage caused by oxidative stress [83]. An increase in the number of M2 macrophages can shift the immunological response through anti-inflammatory cytokines production and reactive oxygen species inhibition [84]. Zinc and doxycycline can induce an M1 to M2 subtype polarization [84] by using an IL-4-dependent pathway [85], which, in turn, suggests zinc immunomodulatory capability [71]. This modulation results in a lack of fibrotic capsule development or low thickness around the Zn-Nm. Madden et al. (2010) [86] fabricated poly (2-hydroxyethyl methacrylate-comethacrylic acid) (pHEMA-*co*-MAA) hydrogel scaffolds, too, obtaining minimal fibrotic response and maximum vascularization, which combined with an increase in the number of M2 phenotype macrophage cells.

**Figure 4:**
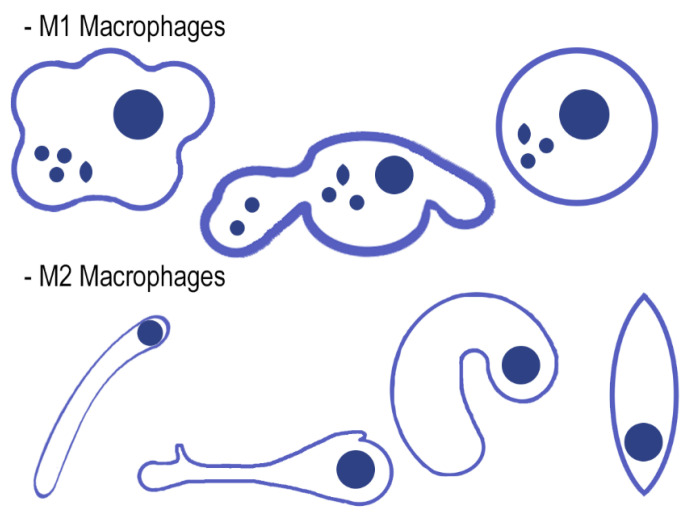
M2 show an elongated shape and long filopodia. M1 are nearly round or irregularly spherical with more lamellipodia [88].

There are theories suggesting that differences in the chemical composition of matrices can control the macrophages’ phenotype ratio (M1/M2). This ratio has been described as a crucial factor for the determination of membrane integration. Changes in this ratio or an increase in the number of M2 macrophages can be a potential strategy to protect the biomaterial [80]. The investigation and further consideration of the mechanisms that guide the polarization of the macrophages and the change between M1 and M2 states are of current interest and might lead to novel therapeutic approaches [87]. The morphology and shape of macrophages have been defined as key factors in the macrophages’ phenotypic polarization modulation [88,89]. Pro-inflammatory M1 and anti-inflammatory M2 macrophages indicate two poles of an overlapping cellular activities continuum; their morphology can be employed to define polarization outcomes, as it has been previously reported [61,81]. An example of their morphology can be observed in Figure 4.

The M1/M2 ratio obtained its lowest value in the groups where Zn-Nm and Dox-Nm were assessed [10]. Zinc is defined as an essential trace element, and it can be found in some key transcription factors and enzymes. It is classified as a key part in the development of the immune system. The presence of zinc results in an improvement of anti-inflammatory cytokines expression and environment maintenance [71]. In particular, it can induce monocytes differentiation to macrophages and increase the release of pro-inflammatory cytokines, including IL–1, IL–6, and tumor necrosis factor (TNF)α–IL1β [71]. Furthermore, macrophages’ spread and attachment can be assisted by porous structures: subsequently, mechanical and physical signals of the porous surface, about 6.99 µm in Zn-Nm [63], can be transformed in biological signals, modulating local macrophage polarization and the microenvironment [71].

In the case of soft tissue augmentation, M1 are essential in the reorganization of damaged tissue through the activation of some enzymes and immunostimulatory cytokines (TNFα–IL1β and IL–6) selection [84], subsequently enhancing inflammation, fibrosis, and tissue injury [81]. It is considered that HOOC-Si-Membranes-treated animals (without Zn or Dox presence) with higher M1/M2 ratios will follow a chronic pro-inflammatory tissue reaction, leading to negative effects in the remodeling of tissue, including fibrous encapsulation, as it has been indicated previously [90].

## 4. Hydrogels, Promising Breakthrough Technology for Soft Tissue Surgery. Mechanically Enhanced Semi-Interpenetrating Polymer Network (IPN) Hydrogel

Recently, hydrogels have been used as scaffolds in the revolutionary tissue engineering field to guide the growth of new tissues. The evolution of hydrogels’ (both biodegradable and non-biodegradable) design and application has enhanced their potential in the biomedical field. Polymer hydrogels are one of the most feasible classes of biomaterials for creating 3D porous scaffolds, as they can mimic the extracellular matrix (ECM) and modulate cell fate. Meanwhile, hydrogel networks can also facilitate matrix remodeling, cell migration, and cell adhesion in a 3D environment, which are required for the normal development of functional tissues [43]. Hydrogels have also allowed the development of new exciting advances in tissue engineering applications and controlled drug delivery [2]. They can be classified according to the synthetic methods, physical properties, polymer source, ionic charge, degradation rate, and cross-linking types. A variety of polymers have been used to synthesize hydrogels, which include natural polymer hydrogels, synthetic polymer hydrogels, and derivative hydrogels [43].

**Figure 5:**
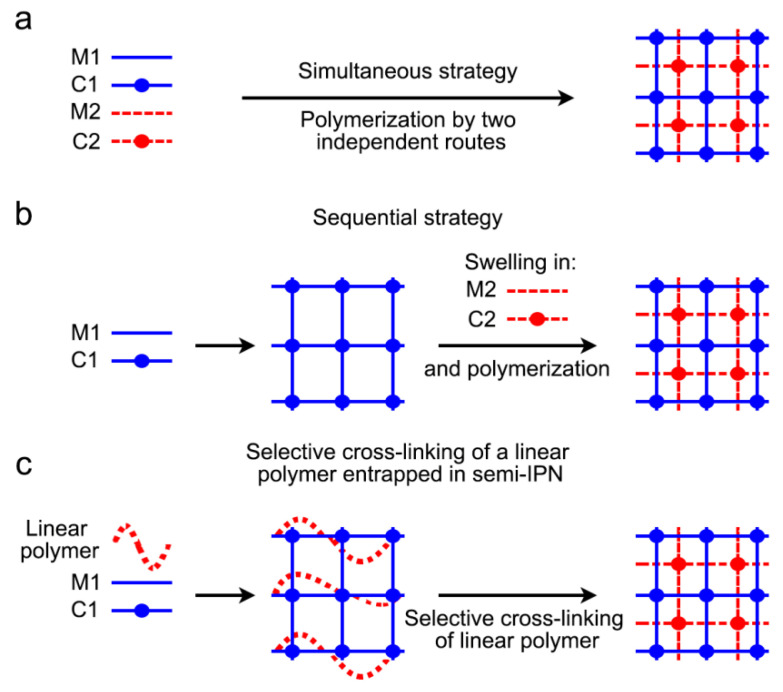
From a synthetic standpoint, interpenetrating polymer networks (IPNs) come in two varieties: (1) sequential IPN, in which one network is swollen with the monomeric composition of the second one, and then the polymerization of the second network is carried out, (2) simultaneous IPN, in which both networks are synthesized at the same time by non-interfering routes. When one of the two copolymers has a linear structure instead of a cross-linked one, a semi-IPN is obtained. Adapted from Dragan (2014) [91] [(**a**) simultaneous strategy; (**b**) sequential strategy; (**c**) selective cross-linking of a linear polymer entrapped in a semi-IPN].

Hydrogels are defined as three-dimensional and hydrophilic networks that are able to withhold huge amounts of water or other biological fluids. They exhibit a rubbery and soft consistency, similar to that of living tissues. Hydrogels have been previously used in Dentistry, as self-inflating soft tissue expanders that offer enough *de novo* soft tissue for vertical bone augmentations and cause a minimal amount of complications. These self-inflating soft tissue expanders are formed by an osmotic active hydrogel, methylmethacrylate, and vinyl pyrrolidone, which offer the possibility of controlling the expansion and volume and/or speed [4]. Any change in environmental conditions (i.e., temperature, pH, and ionic strength) can disintegrate hydrogels formed solely by physical interactions (physical hydrogels). On the other hand, permanent or chemical hydrogels are formed by covalently cross-linked networks. The enhancement of the swelling/deswelling response and the mechanical strength has been achieved due to the design of multicomponent networks as interpenetrating polymer networks (IPNs) [91]. These IPNs have been defined as copolymers “alloys”, as one of the polymers requires the presence of the other in order to be synthesized and/or cross-linked without any covalent bonds between them. Therefore, to separate the copolymers from an IPN, the covalent bonds must be broken. In the case of copolymers with identical structures (linear or cross-linked), a homo-IPN is achieved (Figure 5). It has also been described that a combination of techniques can be performed to improve the mechanical properties of the scaffold. For example, electrospinning together with 3D printing has been previously tested to increase the mechanical properties of the scaffolds [92].

Gels’ responsivity can be altered by both a second network or by the polymer linearly entrapped in a semi-IPN. Semi-IPN hydrogels’ responsiveness (deswelling/reswelling kinetics) is normally much faster than that of single-network hydrogels (both in hetero-semi-IPN and homo-semi-IPN). It has been observed that the majority of synthetic polymers-based IPN composite hydrogels can react to two or three external stimuli. Single-network hydrogels display slow response at swelling and weak mechanical properties [91]. In recent years, the potential of mechanically enhanced IPN hydrogels as “double networks” has attracted a great deal of attention for the design of synthetic biomaterials, which is mostly due to its ability to mimic natural cartilage [93,94,95].

When growth factors are added to the hydrogels, in newly formed tissues, they can act directly in cells’ differentiation and development. Hydrogels support cell migration, high water content, angiogenesis, and rapid nutrient diffusion. Their biochemical similarity with some connective tissues’ highly hydrated components has increased the interest in the study of hydrogel scaffolds for the engineering of connective tissues. Some requirements should be preserved, including their optimum pore size for neovascularization (5 μm) and fibroblast ingrowth (5–15 μm). It is also important that all cells are located in a 200 μm radius from blood supply, as stated before, to ensure the mass transfer of oxygen and nutrients [2]. The concept of “smart” hydrogels refers to scaffolds that can alter their volume/shape when they face small environmental changes [91]. They have been applied in biomedical procedures, such as tissue engineering, wound dressing, or drug delivery. Therefore, due to the optimal results achieved in bone regeneration with our nanostructured polymeric membranes manufactured by electrospinning [10,63], our next goal is synthesizing mechanically enhanced semi-IPN hydrogels (linear/crosslinked). The same chemical composition (high concentration of OH and COOH groups) and functionalization (Zn^2+^, Ca^2+^ or doxycycline) as the electrospinning-processed membranes will be preserved in order to design biocompatible soft materials that are capable of mimicking the physical and chemical properties of the oral soft tissues.

## 5. Protocol of Research on Matrices for Soft Tissue Augmentation

Any matrix employed for soft tissue augmentation to be tested will be comprised in this section. Regardless of the matrix nature or origin, a precise description of the technique that is needed to fabricate the material that will be employed is required. Both raw materials and reagents as well as chemical and engineering procedures involved in the production of the final biomaterial must be included. 

### 5.1. Description of the Matrix and Sample Preparation

In our case, mechanically enhanced IPN hydrogels only based on synthetic polymers are currently being obtained and will be proposed for soft tissue augmentation. Fourier transform infrared (FTIR), scanning electron microscopy (SEM), differential calorimetry (DSC), and equilibrium swelling were applied previously to characterize semi-IPN.

Covalent and ionic bonds stabilize different chains (networks), contributing to the mechanical strength increase and thus to the ionic and pH strength reversible responsiveness [91]. When opposite charges acquire a certain ratio between them, they can form polyion complexes with a multifunctional role. Our currently goal is focused on the design and optimization of semi-IPN matrices. They are based on the blend [(MMA)_1_-co-(HEMA)_1_/(MA)_3_-co-(HEA)_2_] as a lineal copolymer that is interlaced with a highly hydrophilic cross-linked network formed by the in situ polymerization of methacrylic acid (MAA), 2–hydroxyethyl methacrylate (HEMA), and ethylene glycol dimethacrylate (EDMA). The semi-IPN also includes SiO_2_ nanoparticles (NPs–SiO_2_) at different wt %. NPs–SiO_2_ will be homogenously dispersed and trapped in the whole matrix volume, forming a solid solution (composite). Besides, silicon incorporation enhances osteoblast-like cell activity [96]. The degree of swelling and the mechanical properties of our semi-IPN matrices will be studied and characterized in the pH range 5.5–7.5 in the presence of different concentrations of Zn and docycycline (Dox); carboxyl groups are able to bind divalent ions by complexation, and Dox molecules are able to bind divalent ions by acid-basic interactions, which allows the functionalization of the semi-IPN with zinc and Dox. On the other hand, Dox-HOOC-Si-Matrices and Zn-HOOC-Si-Matrices have been demonstrated to stimulate mesenchymal stem cells (pluripotent cells that are able to differentiate into osteoblasts) equally [10]. 

Adequate preparation of samples is a mandatory step to undertake both in vitro and in vivo assays. Matrix discs will be created with a sharp, cylindrical, sterile surgical punch. The samples diameter (6.0 ± 0.1 mm) will be controlled with a sterile sliding caliper. Afterwards, a sterile 0.9% sodium chloride solution should be applied to hydrate the samples for 5 min [62].

### 5.2. Cell Culture Fibroblasts, Osteoblasts, Endothelial Cells, Keratinocytes, and Cell Morphology

Human umbilical vein endothelial cells (HUVEC), human osteoblast-like (HOB), human oral keratinocytes (HOK), and human gingival fibroblasts (HGF) are used in the experiments. Cells should be cultured at 37 °C in an incubator with 95% air and 5% CO_2_. HGF are normally cultured in Stromal Cell Growth Medium with 10% fetal calf serum (FCS), 1 ng/mL basic fibroblast growth factor, and a 1% penicillin-streptomycin-neomycin antibiotic mixture. HOB will be seeded in a solution of Dulbecco’s modified Eagle’s medium (DMEM) with 10% FCS, 1% L-glutamine, and 1% penicillin-streptomycin-neomycin antibiotic mixture. Previously, osteocalcin (labeled streptavidin-biotin/horseradish peroxidase) and alkaline phosphatase immunohistochemical expression are used to characterize osteoblasts. The culture of HUVECs will be obtained in an endothelial basal medium complemented with 10% FCS, 10 ng/mL epidermal growth factor, 50 µg/mL gentamycin, 50 ng/mL amphotericin B, 12 µg/mL bovine brain extract, and 1 µg/mL hydrocortisone. Finally, HOK will be seeded in keratinocyte growth medium. The medium contains 1 ng/mL epidermal growth factor, Ca^2+^ < 0.1 mM, 1 ng/mL fibroblast growth factor, and insulin, without bovine pituitary extract and without hydrocortisone [62]. HGF cells’ morphology and distribution on the matrix will be assessed with direct fluorescence at 24 h, 5 d, and 14 d. Cell adherence, proliferation, morphology, and interactions with the matrix will be tested via cell culture in 24-well plates (30,000 cells/well) and stained with cytogreen fluorescent dye at different measurement points. They will be observed and photographed with an inverted fluorescence microscope [62].

### 5.3. Cell Viability and Cytotoxicity (LIVE/DEAD^®^ Assay)

Cell viability will be analyzed using several study groups: matrices, negative controls (2% triton X-100 (Sigma, St. Louis, MO, USA)-treated cells), and positive controls (DMEM-cultured cells without matrices). Triton X-100 is a chemical compound with high cytotoxic effects. Cells, after their exposition to each culture condition for 24 h, will be analyzed. Three different techniques are normally employed to evaluate cell viability:

(1) The first is cell death, which is determined by observing nuclear membrane integrity. It is measured by the quantification in the culture media of the liberated deoxyribonucleic acid (DNA). From each sample supernatant, 10 μL aliquots are acquired and diluted in nuclease-free distilled water 10 times. DNA in the medium can be quantified by spectrophotometry in the wavelength range of 260–280 nm. For each experimental group, three independent experiments will be undertaken, and their mean values and standard deviations will be reported. (2) Lactate dehydrogenase (LDH) is an enzyme released in cells with damaged membranes. LDH assay can detect the amount of this enzyme and can be used as a cell death indicator. In each experimental group, cells are incubated with the particles dilutions, using the supernatants to quantify LDH. Particles’ interferences in the measurements are investigated via LDH assay in cell supernatants and transferred to 96-well plates after their centrifugation (10 min, 200 g). Five independent determinations are obtained from each experimental group. (3) The third technique involves examining cell membrane integrity and cytoplasmic esterase function by a fluorescence-based method. The live/dead viability/cytotoxicity kit LIVE/DEAD^®^ commercial kit is recommended to be used. This method relies on the use of calcein-AM, which is a marker that living cells metabolically transform in a green pigment, and ethidium homodimer-1, which stains dead cells nuclei red. Following cells incubation with each biomaterial, supernatants are discarded, and cells are washed with PBS. These cells are incubated for 15 min with the live/dead solution following the manufacturer’s instructions and washed again with PBS. Samples are subsequently studied in a fluorescence microscope. Five images are taken for each experimental condition and processed with *ImageJ* to quantify the number of red (dead) and green (live) cells [63].

### 5.4. Animal Experimentation

A general overview of the most remarkable animal experimentation protocols is shown in Table 1.

#### 5.4.1. Mouse Experimental Design

This methodology was proposed to study experimental soft tissue augmentation in small animals by Pabst et al. (2014) [62]. An ethical committee should approve all animal experiments. In this test, six female euthymic nude mice are used (age 6–8 weeks, weight 26 and 28 g). These mice are acclimated before treatment for 14 d and housed at 22 °C in a 12 h light-dark cycle with food and water *ad libitum*. By using a sodium chloride solution, ketamin and xylazine narcotic mixture (250 µL each per animal) via intraperitoneal injection with a 26-gauge needle, mice are anesthetized before matrix implantation. Matrix will be also harvested after killing mice with an overdose of the narcotic mixture. In each mouse, two different matrices (diameter 8.0 ± 0.1 mm) are implanted subcutaneously right and left alongside the dorsal midline after the first 2 weeks of acclimatization. The mice are killed after 21 d in situ [62]. 

Matrices and their adjacent tissues will be dissected from the back and put for 2 d at room temperature in 10% formalin for tissue histology and sampling. From each specimen, respectively, three microslides from the center and the border areas for each of the four different stainings: hematoxylin and eosin (H&E), toluidine blue (TB) CD31, and Ki-67 have to be prepared. Anti-CD31- and anti-Ki-67-stained frozen sections allow the determination of microvessel and proliferation densities. A monoclonal antibody against CD31 will be used to perform endothelial cells immunohistochemical staining, while a monoclonal antibody against Ki-67 is applied to perform proliferating cells immunohistochemical staining. Antibody binding can be visualized by a three-step staining procedure using a biotinylated polyclonal antirat IgG secondary antibody and the streptavidin horseradish peroxidase reaction together with the DAB staining inmunohistochemistry. For immunohistochemistry negative controls, matrices slides will be stained with the antibody dilution solution without a primary antibody, which is normally used for the attenuation of the primary antibody against Ki-67 and CD31. Finally, a morphometry software must be used [10,62].

#### 5.4.2. Dog Experimental Design

This methodology was proposed to study experimental soft tissue augmentation in large animals by Schmitt et al. (2016) [104]. In the procedure, eight healthy female beagle dogs (age at least 12–18 months) are selected. All animals receive a transponder to ensure their clear allocation to the experimental protocol before the study. General anesthesia is applied before every surgery, and 10–15 min before general anesthesia administration, a pre-treatment sedation (midazolam 0.05–0.1 mg/kg and ketanest 1–2 mg/kg intramuscularly) is used. Thiopental sodium (20 mg/kg body weight) is used to anaesthetize the dogs, and nitrous oxide, oxygen, and isoflurane (1.5–2.0% isoflurane in 2 L/min. oxygen flow) are applied to perform the inhalation. Simple randomization is applied to locate four matrices to either the right or left upper or lower canine. After doing the surgery, the animals receive post-surgical antibiotics for 3 days. Then, the animals should be kept warm and monitored until they are fully recovered. Post-surgical care includes a daily observation, documenting adverse events such as appetite, discomfort, swelling, pain, and bleeding.

Jaws are first sectioned for tissue sampling and histology using a precision saw that allows obtaining small samples from the augmented region of interest (ROI). Samples are immersed in formalin solution to be fixed, dehydrated in alcohol, and embedded in Technovit 9100 New. Using a grinding unit, half of each embedded section is reduced to 25–30 μm. They are subsequently polished, stirred continuously in 10% H_2_O_2_ for 5 min, rinsed under cold running water, dried, and stained with toluidine blue O solution for 15 min. Sections are coated and light cured for 8 min. A specific software is used to analyze digitized histological data and evaluate tissue thickness in the augmented regions of interest (ROIs) in five regions per sample. The half that has not yet been treated from each embedded section is submitted to fixation in a sledge microtome. The sample is sliced into multiple 2 to 4-μm-thick sections, processed, and dried on microscope slides at 57 °C for 12 h. In order to allow a descriptive histological analysis centered in the augmented ROIs connective tissue portions, a Cason staining is performed. Sections are stained for collagen I and VEGF expressions in immunohistochemical analyses with the avidin-biotin complex method. Sections are next subjected to chromogen treatment and then counterstained with hematoxylin, followed by fixation. VEGF and collagen I expression can be quantitatively calculated in the connective tissue areas of those five samples that corresponded to the regions defined for thickness measurements.

## 6. Trends for Future

Our proposal of matrix for soft tissue augmentation biomaterial, following the concept of “structure-related properties” [8] lays on the development of a semi-IPN formed by the blend [(MMA)_1_-co-(HEMA)_1_/(MA)_3_-co-(HEA)_2_] as a lineal copolymer that is interlaced with a cross-linked network by the in situ polymerization of MAA, HEMA, and EDMA in the presence of SiO_2_ nanoparticles. Then, the SiO_2_-semi-IPNs that are swelling with solutions of Zn or doxycycline at different pHs, in order to obtain a functional bioinspired material, is our choice. Additive biomanufacturing offers favorable strategies for soft tissue augmentation engineering. Currently, we cannot find any synthetic biomaterial that mimics the soft tissues structure; so, the proposal of this new biomaterial for soft tissue augmentation implies a step toward the design of the gold standard biomaterial for this purpose. Despite the improvements achieved so far, the development of novel approaches remains at a rapid pace. Multicomponent materials whose individual constituents can organize in an autonomous way into tailored superstructures are proposed in the present manuscript. An interdisciplinary effort is required to exploit our polymeric hybrid materials potential, which is reflected and is based on the interplay between these advanced materials’ performance, structure, processing, and synthesis. Besides fabrication techniques, scaffold design, and biomaterials optimization, standardization is a key step toward their effective implementation in the future. Incomparable to thermoplastics, thermosets, ceramics, and metals, hydrogel-based 3D printing is playing a pivotal role in the design and creation of advanced functional (bio)systems in a customizable manner [43].

## 7. Conclusions

From the present review, it can be concluded that there is a lack of a material that can fulfill the requirements (mainly 3D stability, non-immunogenic, topography ECM mimicking) for being used in oral soft tissue augmentation. That is why, throughout this review, we aimed to determine a new biomaterial to offset the present shortcomings of the current matrices for soft tissue augmentation in the oral environment. Researchers have recently been proposed a novel non-resorbable composite membrane, manufactured via electrospinning with a mixture of (MMA)_1_-co-(HEMA)_1_ and (MA)_3_-co-(HEA)_2_ that has allowed obtaining remarkable in vivo *outcomes*, concerning angiogenesis and immunomodulation throughout macrophages polarization. This same polymer blend is currently being used to synthesize hydrogels, as multicomponent semi-IPNs of a definitive biocompatible and non resorbable matrix.

## Figures and Tables

**Table 1:** Different animal models (dogs, mice, rats, or primates) can be submitted to soft tissue augmentation/experimentation.

ART.	Animals	Sample and Treatment Modalities	Surgeries	Sacrifice	Histological and Histometric Assessment.
Thoma et al. (2011) [97]	Six male large hound type dogs *Age: >2 years* *Weight: 60–70 kg*	36 samples *(six samples per animal; two samples per group)* ***Group A:*** Porcine collagen matrix (CM) *(Geistlich Pharma AG, Wolhusen, Switzerland)* ***Group B:*** Subepitelial connective tissue graft (SCTG) ***Group C:*** Sham-operated site *(Control)*	All mandibular P2, P4, and the distal roots of M1 were extracted on both sides of the mandible and the buccal plate of the extraction sites was removed. After a healing period of 2 months, full-thickness mucoperiosteal flaps were elevated to receive the treatment.	At 28 days (*n* = 3) and 84 days (*n* = 3) following soft tissue augmentation surgery, euthanasia was performed on all animals.	Qualitative analysis with a stereoscope: old bone, newly formed bone, non-mineralized bone, collagen matrix, vascularization of the matrix, tissue integration, and inflammatory reaction. Computer-assisted histomorphometric measurements: augmented ridge width in four different levels 1.5, 3.5, 5.5, and 7.5 mm below the crest, including measurements of native bone, newly formed bone, and non-mineralized tissue.
Pabst et al. (2014) [62]	Six female euthymic nude mice *Age: 6–8 weeks* *Weight: 26–28 g*	12 samples *(two samples per animal)* ***Treatment:*** Porcine-derived collagen matrix *(Mucoderm, Botiss Dental, Berlin, Germany)*	Two matrices were implanted subcutaneously right and left alongside the dorsal midline of each mouse.	After 21 days (*n* = 6), the mice were sacrificed.	Analysis with a stereoscope: Three microobjects were obtained from each sample for each of the different stains Hematoxylin and eosin (H&E) stained cross-sections for histological assessment. CD31 immunohistochemical staining for microvessel detection and proliferation densities. Ki-67 immunohistochemical staining for the detection of proliferating cells. Antibody binding using three-step staining procedure.
Rothamel et al. (2014) [98]	Forty albino rats of the Wistar strain *Age: 3 +/− 0.5 months* *Weight: 350 +/− 21 g*	160 samples *(four samples per animal: one per group)* **Native (ND)** (Mucoderm^®^ Prototype) ***Specifically defatted (DD)*** ***Ethylene dioxide cross-linked (ECL)*** ***Dehydrothermally cross-linked (DCL)*** dermis collagen *(AAP/Botiss Biomaterials, Berlin, Germany)*	A skin incision was made paramedian along the vertebral column followed by the separation of four unconnected subcutaneous pouches. The membranes were randomly allocated in the resulting 160 pouches.	At 1 (*n* = 5), 2 (*n* = 5), 4 (*n* = 5), 8 (*n* = 5), and 12 (*n* = 5) weeks, animals were sacrificed.	Histomorphometricanalysis: For image acquisition, a color CCD camera was mounted on a binocular light microscope. Digital images were evaluated using an imaging program to evaluate: Membrane thickness: linearly at 12 fields selected at random. Descriptive parameters: vascularization of the membrane body, tissue integration, and foreign body reaction and cell invasion.
Ferrantino et al. (2016) [99]	Six mature beagle dogs *Weight: 11.6–14.5 kg*	***Split-mouth study***; 12 samples *(2 samples per animal; one per group)* ***Test group:*** VCMX *(Fibro-Gide^®^ prototype, Geistlich Pharma AG, Wolhusen, Switzerland)* ***Control group***: no treatment	The study was divided into three phases: surgical extraction of six maxillary premolars per animal, surgical matrix placement with full thickness flap and either a VCMX in contact with the bone or the flap repositioned without the use of a biomaterial (sham group), and evaluation prior to sacrifice.	At incremental time points, including day 0 (*n* = 1) and 4 (*n* = 1), 7 (*n* = 1), 15 (*n* = 1), 30 (*n* = 1), and 90 (*n* = 1) days after matrix placement, the canines were sacrificed.	Prior to microscopic examination, the samples were cut into slices and stained with different stains (hematoxylin/eosin, basic fuchsin, and toluidine blue; double stained with toluidine blue and basic fuchsin). Microscopy examination: tissue integration process, cell and blood vessel invasion, and new collagen formation.
Herford et al. (2018) [100]	Six skeletally mature male nonhuman primates (Rhesus macaques)	***This study also performs a combination part with bone guided regeneration (BGR)*** ***Group A:*** Volume stable porcine collagen matrix *(Fibro-gide^®^, Geistlich Pharma AG, Wolhusen, Switzerland)* ***Group B:*** SCTG ***Group C:*** No treatment.	Surgery 1: Tooth extraction and creation of a bony defect. Surgery 2: Soft tissue augmentation procedures with partial-thickness flaps. Surgery 3: Implant placement.	No sacrifice. One month post-implant placement, the soft tissue surrounding the implant was biopsied.	***Soft Tissue Analysis (In Vivo):*** Periodontal probing, shear modulus elasticity, and volumetric analysis. ***Soft Tissue Analysis (In Vitro):*** Qualitative analysis with a stereoscope: Vascularization of the matrix, collagen matrix evaluation, inflammation, and tissue integration. Histomorphometric assessment: Images were captured via digital camera and light microscope; the images were evaluated using analysis software.
Seo et al. (2019) [101]	Four male beagle dogs *Age: 18–24 months* *Weight: approx. 15 kg*	***Split-mouth study***; 24 samples *(6 samples per animal; 3 sample per side)* ***Test group:*** BGR. + guided tissue regeneration (GTR) with collagen matrix *(Collagen Graft, Genoss, Suwon, Korea)*. ***Control group:*** No additional application of collagen matrix layer	A standardized bony defect was surgically created bilaterally. R.O.G procedures were performed. In the test group, an additional collagen matrix was applied over the collagen membrane; subsequently, a periosteal releasing incision was made to allow advancement of the mucoperiosteal flap.	At 8 weeks of healing, all animals were euthanized.	Microcomputed tomography analysis: The scanned dataset was processed in DICOM format and reconstructed with three-dimensional software in order to measure thickness of the soft-tissue layer. Microscopy examination: Using an image analysis software. Outcomes measured in the region of interest (ROI); thickness of the soft-tissue layer, thicknesses of the membrane complex including the dense connective tissue above the membrane, the membrane, and the dense connective tissue below the membrane, proportions of new bone formation, remaining bone material and connective tissue.
Song et al. (2019) [102]	Six beagle dogs *Age: 12–15 months* *Weight: 10–15 kg*	***Split-mouth study***; 12 samples *(2 per animal)* ***BCCM:*** Bovine-derived CCM *(Genoss, Suwon, Republic of Korea)* ***PCCM:*** Porcine-derived CCM *(SK Bioland, Cheongju, Republic of Korea)* ***B-control group:*** Negative control site at contralateral side of BCCM group. ***P-control group:*** Negative control site at contralateral side of PCCM group.	***Tunnel technique*** was made on an attached gingiva underneath the mesiolabial side of the first incisor area to distolabial side of the third incisor area.	At 12 weeks after the operation, the animals were sacrificed.	Microscopy examination: Analyzed with a computer software. Parameters measured: Horizontal thickness (mean thickness of total soft tissue, mean thickness of total connective tissue, and mean thickness of dense connective tissue) and number of rete pegs (rete pegs underneath the keratinized epithelium and rete pegs underneath the sulcular epithelium and junctional epithelium). Volumetric analysis: Dental impressions at all five time points were obtained and digitized using a dental scanner. The resulting STL files were subsequently analyzed using software.
Caballé-Serrano et al. (2019) [103]	Six beagle dogs *Weight 11.6–14.5 kg*	***Split-mouth study***; 12 samples *(2 samples per animal; one per group)* ***Test group:*** VCMX *(Fibro-Gide^®^ prototype, Geistlich Pharma AG, Wolhusen, Switzerland)* ***Control group:*** No treatment	Six maxillary premolars were atraumatically extracted to create two edentulous spaces in each dog. After 90 days, a full thickness flap was elevated, and either a VCMX in contact with the bone was placed, or the flap was repositioned without the use of a biomaterial (sham group).	At incremental time points, including day 0 (*n* = 1), 4 (*n* = 1), 7 (*n* = 1), 15 (*n* = 1), 30 (*n* = 1); and 90 (*n* = 1) days after matrix placement, the canines were sacrificed.	Scanning Electron Microscopy examination MAC387 Immunohistochemistry: To visualize cells that belong to the inflammatory linage derived from hematopoietic stem cells such as granulocytes, monocytes, and macrophages. PCNA Immunohistochemistry: To visualize cells that were in a proliferative phase. Quantitative Analysis CD86 Immunohistochemistry TGM2 Immunohistochemistry: To stain blood vessels.
Schmitt et al. (2019) [69]	Eight healthy female beagle dogs *Age: 12–18 months*	***Randomized split-mouth study:*** 16 samples *(2 samples per animal; one per group)* ***Test group:*** Natural porcine 3D collagen matrix *(CM, mucoderm^®^, Botiss Biomaterials GmbH)* ***Control group***: SCTG *(control group)* from the palate.	The two treatments (CM and SCTG) were allocated to either the right or left upper canine by simple randomization. The ***tunnel technique*** was performed in an extent that the soft tissue graft could be inserted stable without the need of any further fixation.	After 10 months, the animals were sacrificed.	Histomorphometrically measured connective tissue thickness (CTT) in mm. Descriptive histological analyses, Immunohistochemical analyses, and Immunohistological quantification: Vascular endothelial growth factor (VEGF): With the VEGF quantification, differences between groups concerning tissue vascularization should be detected (e.g., due to ongoing integration/degradation processes or inflammation). Collagen I expression to qualify the augmented soft tissues. Inter-group differences of the collagen density should be detected.

## Abbreviations

BMP	Bone morphogenetic protein
CAD	Computer-aided design
CAM	Computer-aided manufacturing
DMEM	Dulbecco’s modified Eagle’s medium
DNA	Deoxyribonucleic acid
DSC	Differential calorimetry
ECM	Extracellular matrix
FCS	Fetal calf serum
FTIR	Fourier transform infrared
H&E	Hematoxylin and eosin
HGF	Human gingival fibroblasts
HOB	Human osteoblast-like
HOK	Human oral keratinocytes
HUVEC	Human umbilical vein endothelial cells
IFN-γ	Interferon gamma
IL	Interleukin
IPN	Interpenetrating polymer network
LDH	Lactate dehydrogenase
MMP	Metalloproteinase
MNGC	Multinucleated giant cell
Nm	Nanostructured membranes silica loaded
(Zn-Nm)	Nanostructured membranes silica loaded doped with Zinc
(Dox-Nm)	Nanostructured membranes silica loaded doped with Doxycycline
NPs	Nanoparticles
PCL	Polycaprolactone or poly(ε-caprolactone)
PGA	Poly(glycolic acid)
pHEMA-co-MAA	2-hydroxyethyl methacrylate-co-methacrylic acid
PLA	Poly(lactic acid), polylactic acid or polylactide
PLGA	Poly(lactic-co-glycolic) acid
ROI	Region of interest
SEM	Scanning electron microscopy
TB	Toluidine blue
TGF-β	Transforming growth factor beta
TNF-α	Tumoral necrosis factor alpha
VEGF	Vascular endothelial growth factor
